# Supplementary material for: Integrative multi-omic analysis identifies key transcription factors and target proteins in renal cell carcinoma and its subtypes
Source: bioRxiv. 2025 Nov 26:2025.11.23.690024. Preprint. [Version 1] doi: 10.1101/2025.11.23.690024 (PMC12703995; doi:10.1101/2025.11.23.690024)
Supplement: Supplement 2 [file media-2.docx]

**Supplementary Figures**


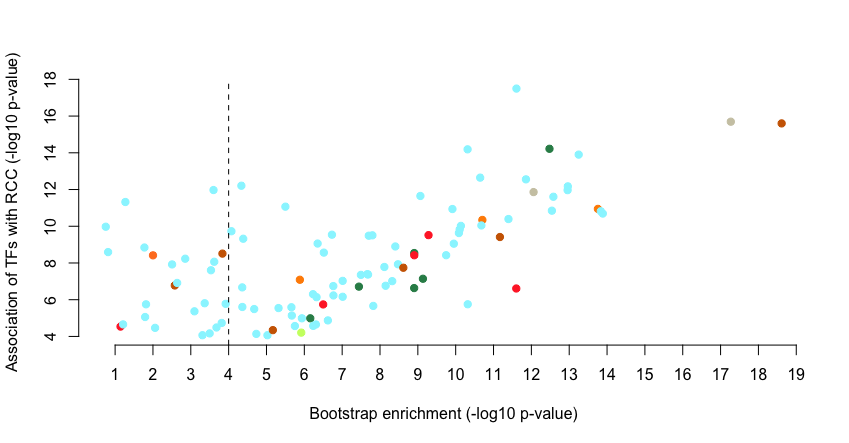


**LMM vs Bootstrap**

**LMM vs S-LDSC**


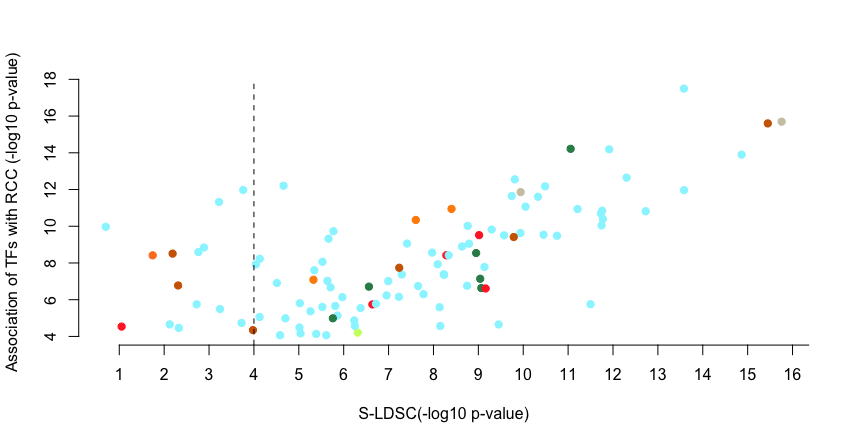


**Figure S1:** Comparison of association of TFs with RCC (colored by cell lines) using the mixed model-based approach (See **Methods**) against that with Bootstrap enrichment (upper panel) and stratified LD score regression (S-LDSC; lower panel).


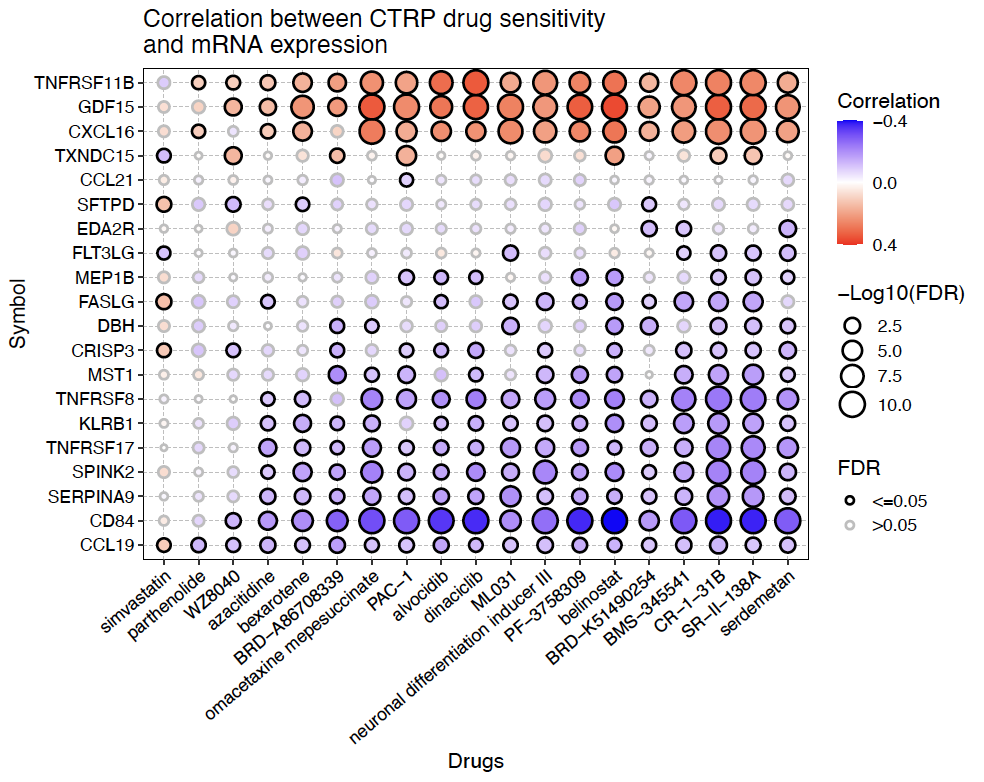


**Figure S2:** Bubble plot showing the correlation between gene expressions corresponding to 21 proteins trans-associated at least 10 TFs and IC50 for different drugs. P-values for the test of correlation adjusted for false discovery rate. Data and plot generated from Gene-set Cancer Analysis web portal(GSCA: [www.guolab.wchscu.cn/GSCA](http://www.guolab.wchscu.cn/GSCA))
